# Population-Wide Failure to Breed in the Clark’s Nutcracker (*Nucifraga columbiana*)

**DOI:** 10.1371/journal.pone.0123917

**Published:** 2015-05-13

**Authors:** Taza D. Schaming

**Affiliations:** Department of Natural Resources, Cornell Lab of Ornithology, Cornell University, Ithaca, New York, United States of America; Hungarian Academy of Sciences, HUNGARY

## Abstract

In highly variable environments, conditions can be so stressful in some years that entire populations forgo reproduction in favor of higher likelihood of surviving to breed in future years. In two out of five years, Clark’s nutcrackers (*Nucifraga Columbiana*) in the Greater Yellowstone Ecosystem exhibited population-wide failure to breed. Clark’s nutcrackers at the study site experienced substantial interannual differences in food availability and weather conditions, and the two nonbreeding years corresponded with low whitebark pine (*Pinus albicaulis*) cone crops the previous autumn (≤ an average of 8 ± 2 cones per tree versus ≥ an average of 20 ± 2 cones per tree during breeding years) and high snowpack in early spring (≥ 61.2 ± 5.5 cm versus ≤ 51.9 ± 4.4 cm during breeding years). The average adult body condition index during the breeding season was significantly lower in 2011 (-1.5 ± 1.1), a nonbreeding year, as compared to 2012 (6.2 ± 2.0), a breeding year. The environmental cues available to the birds prior to breeding, specifically availability of cached whitebark pine seeds, may have allowed them to predict that breeding conditions would be poor, leading to the decision to skip breeding. Alternatively, the Clark’s nutcrackers may have had such low body energy stores that they chose not to or were unable to breed. Breeding plasticity would allow Clark’s nutcrackers to exploit an unpredictable environment. However, if large-scale mortality of whitebark pines is leading to an increase in the number of nonbreeding years, there could be serious population-level and ecosystem-wide consequences.

## Introduction

The ability to modify reproduction as a function of environmental conditions better allows species to exploit variable, unpredictable environments [1,2]. Life-history theory suggests individuals should adopt a bet hedging strategy to maximize fitness in variable environments in which they reduce annual reproduction in poor years to increase survival and lifetime reproductive success [3,4]. Foregoing a breeding season may be an adaptive plastic response, favored when the value of immediate reproduction is low compared to the value of survival and future reproductive opportunities [5,6]. In highly variable environments, conditions can lead to such poor potential for breeding that all individuals in a population forgo reproduction in favor of higher likelihood of surviving to breed in future years. Although the proximate mechanisms remain poorly understood [7], population-wide failure to breed can come about in two non-mutually exclusive ways. First, if, prior to the onset of breeding, reliable cues are available indicating that environmental quality will adversely affect that year’s reproductive success, individuals could use these cues to adaptively modify reproductive effort, potentially foregoing breeding all together [8,9]. Second, if prebreeding food supplies or weather are unfavorable, the entire population may have such low body energy stores as compared to body size that it is beneficial to skip a year before attempting to breed; this may either be an adaptation or a constraint [10,11].

Overall, conditions favoring population-wide failure to breed should be relatively uncommon, occurring mainly in ecosystems with extreme annual variation in resources (e.g. food) and weather, such as arid systems or montane regions [12–14]. Montane areas are therefore ideal locations for investigating behavioral responses that permit or limit reproductive success in highly variable environments [15]. Previous research suggests that population-wide failure to breed is primarily correlated with low food availability or precipitation (drought, high rain or snow) which decreases food supplies [12–14].

With climate change, increased environmental disturbance, landscape change, and declining forest health, instances of failing to breed may become increasingly common and may represent a potential cause of population decline and local extinction. Habitat specialists in particular may be vulnerable to environmental change if they do not adapt. This places a premium on understanding the contexts and causes of the decision to forego breeding for populations under threat of becoming endangered or at risk of extinction.

The Clark’s nutcracker is a long-lived, facultative partial migrant at high altitudes in the montane ecosystems of the western U.S. and Canada [16,17]. They specialize on seeds of masting conifer species, and rely on cached seeds for both overwinter survival and breeding [17,18]. They are unusual in that they primarily feed their young seeds cached the previous autumn [18]. Thus, they have unusually accurate information about spring food supplies at the time when they would initiate breeding. It is not clear how long Clark’s nutcracker spatial memory of seed cache locations lasts. Field observations suggest Clark’s nutcrackers remember the location of cache sites for seven to nine months [19]. Laboratory experiments showed that Clark’s nutcrackers begin to forget the locations between 183 and 285 days [19]. After 285 days, many remaining seeds will have germinated, spoiled or been robbed by other animals, so it is unlikely caches would continue to be available for multiple years [19]. Therefore, caches from years with high cone crops would not supplement the diet during low cone crop years. Clark’s nutcracker populations are highly sensitive to variation in the annual cone crop of conifers such as whitebark pine (*Pinus albicaulis*), limber pine (*P*. *flexilis*) and ponderosa pine (*P*. *ponderosa*); they have been reported to irrupt during years of cone crop failure [20,21].

Ecosystems throughout the Clark’s nutcracker range are currently under threat due to decades of fire suppression, widespread infection of five-needled pines by the non-native fungal pathogen *Cronartium ribicola*, which causes white pine blister rust, and outbreaks of mountain pine beetles (*Dendroctonus ponderosae*) [22]. Despite the Clark’s nutcrackers’ capacity for wide ranging movement, evidence suggests several populations are declining, including those in “pristine” environments such as Glacier National Park in Montana and the Cascade Mountains of Washington [23].

This study is based on five years of field work in the Greater Yellowstone Ecosystem. It involved documenting population-wide breeding effort in a relatively robust population of Clark’s nutcrackers in a community where the whitebark pine trees they depend upon for food are in decline [24]. Previously, all that was known of Clark’s nutcracker breeding biology was based on monitoring relatively few nests (n = 16 nests from two different studies [18,25]), and few accounts of fledgling observations (e.g. [26,27]). Despite the low number of nests monitored, some previous studies have suggested that Clark’s nutcrackers may forego breeding in years with low food [18,26].

Clark’s nutcrackers are a keystone species in western North America because they play an important role in forest regeneration and seed dispersal for at least ten conifer species (see references within [28]). Whitebark pine, a keystone species and a candidate species under the Endangered Species Act, is an obligate mutualist of Clark’s nutcrackers because it germinates almost exclusively from Clark’s nutcracker seed caches [28–30]. Understanding causes of Clark’s nutcracker failure to breed provides important information relevant to their conservation, as well as conservation of the fragile high elevation ecosystems they inhabit. These ecosystems provide essential services, including retention of snowpack critical for maintaining water supplies for much of the western U.S. [31]. My primary objective in this paper is to evaluate the conditions which may contribute to Clark’s nutcrackers’ population-wide failure to breed.

## Methods

### Ethics statement

I captured and handled all birds according to Animal Care Protocol guidelines approved by Cornell University. This research was approved by the Cornell University Institutional Animal Care and Use Committee (protocol # 2008–0176). I banded under U.S. Fish and Wildlife Permit # 23533, and Wyoming Game and Fish Chapter 33 Permit # 695. I conducted all field work under U.S. Forest Service Special-Use Authorization # JAC747002 (2009–2013) and Grand Teton National Park Scientific Research and Collecting Permit #’s GRTE-2011-SCI-0052 and GRTE-2012-SCI-0069.

### Field methodology

#### Study site

**Fig 1:**
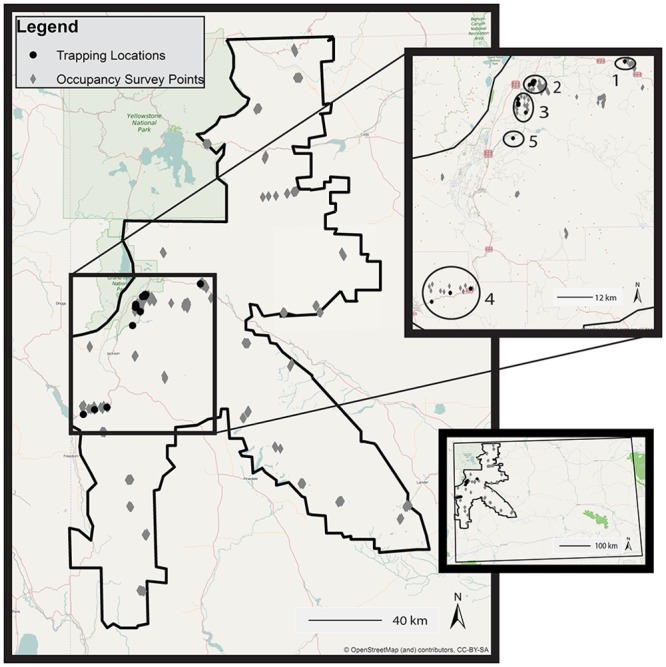
Study area in the Greater Yellowstone Ecosystem. The study area is outlined in black. I spent the majority of time in the field trapping and radio tracking Clark’s nutcrackers, and hiking to and conducting occupancy surveys. The top inset map delineates the five trapping locations. The bottom inset map depicts the study area within the state of Wyoming. (OpenStreetMap basemap: http://www.openstreetmap.org/copyright [32].)

I documented Clark’s nutcracker breeding activity between 2009 and 2013 in the Greater Yellowstone Ecosystem, primarily in Bridger Teton and Shoshone National Forests, and Grand Teton National Park (25,050 km^2^; bounded by 45°00’01” N north, 42°09’14” N south, 111°02’56”W west, and 108°42’55”W east; Fig 1). The forested habitat primarily consists of whitebark pine, limber pine, Douglas-fir (*Pseudotsuga menziesii*), lodgepole pine (*Pinus contorta*), Engelmann spruce (*Picea englemannii*), and subalpine fir (*Abies lasiocarpa*), intermixed with sagebrush (*Artemesia tridentata*)—grass open areas, high mountain meadows and rocky outcroppings.

#### Seasonal boundaries

As no Clark’s Nutcrackers bred in 2011, I based seasonal boundaries on breeding years 2010 and 2012. Based on my observations of Clark’s Nutcracker behavior, the prebreeding season ranged from January 15, the first date I trapped Clark’s nutcrackers, through March 4. The breeding season is considered March 5, the first date in any year a Clark’s nutcracker was seen building a nest, through June 15, the last date a nestling was observed on a nest.

#### Determination of breeding versus nonbreeding years

To evaluate breeding status and breeding activity of individuals and within the population, I used trapping, radio tracking, surveying, and documentation of fledglings and older young of the year. First, I examined each trapped bird to determine if a brood patch was present. Second, at all times while in the field, I documented all breeding activity of radio tagged and unbanded Clark’s nutcrackers, including courtship behavior (Table 1), nest building, and nest attendance. Third, I documented all observed fledglings and older young of the year at all times while in the field.

**Table 1:** Courtship Behavior.

ACTIVITIES	Reference
Bowing	[17,18]
Tail wagging	T. D. Schaming, personal observation
Opening and closing bill while pumping legs	T. D. Schaming, personal observation
Neck stretching	[17,18]
Hopping close together on branch with bills open	T. D. Schaming, personal observation
Wild, rapid flights; chasing, flying directly at one another, and making direct contact in air while both flap their wings	[18,29], T. D. Schaming, personal observation
One crouching, stretching out and fluttering wings, other feeding	[17,18]
Carrying sticks while performing other courtship displays	[17,18]
**CALLS**	
Courtship begging	[17,18]
Musical or chirrup	[17,18]
Hiccup	[17]
Crackle- and- whistle or crackle	[17,18]
Pop-click	[17]
Bullfrog or croaking	[17,18]

Courtship behavior includes one or a combination of the following activities and/or calls.

The easiest way to document breeding in the population is to document observed fledglings. Fledglings are easy to differentiate from adults: they have a noticeably shorter beak and shorter tail; no white on their face, around their bill and eyes; a duller body, wings and tail, with more brown—grey plumage; and when recently fledged, pink spots below their eyes, a red mouth, and grey legs and irides [17]. The young birds are also conspicuous because of their loud, regular begging calls [18,33], and their distinctive flying, landing, foraging and social behaviors (T. D. Schaming personal observation). During the breeding and post-breeding seasons, fledglings (fledged at the site) and/or older young of the year (either fledged at the site or dispersed in from outside the study area, appearing in August or later) were observable. Fledglings are readily recognizable in May through July, and older young of the year are discernible into the autumn. Years in which I observed brood patches, nesting activity and/or presence of fledglings were considered breeding years.

#### Capture and marking

Trapping sites were located within the same three general locations in all years. The first was in high-elevation whitebark pine habitat with some subalpine fir (2,659–2,757 m). The second was in mid-elevation lodgepole pine habitat with some Douglas-fir and Engelmann spruce (2,187–2,265 m), and the third was in mid-elevation Douglas-fir habitat with some subalpine fir and limber pine, and Englemann spruce—lodgepole pine habitat (2,131–2,259 m; Fig 1). In 2011, I attempted to trap at a fourth location in low- to mid-elevation Douglas-fir habitat with limber pine and lodgepole pine (1,763–2,208 m), at which I never observed a Clark’s nutcracker at or near the trapping sites. In 2011, I also trapped at a fifth location in a residential area (2097 m), at which I banded, but did not radio tag any birds. I chose the trapping site locations because they represent all possible conifer habitats, and were accessible, within forty five minutes from the nearest parking area.

At each of the trapping sites, I baited the Clark’s nutcrackers with beef suet and trapped adults in mist nets or bow nets. I weighed (g, 1 decimal place) and measured (culmen (tip of the upper mandible to the first feathers; mm, 1 decimal place), tarsus (bent right leg; mm, 1 decimal place), wing chord (natural curvature; mm, 0 decimal places), and tail (base of tail to tip of longest feather, natural curvature; mm, 0 decimal places)) adults to determine body condition index. I banded each with a U.S. Fish and Wildlife Service aluminum band and three colored leg bands. I determined the amount of body fat with a furcular fat score of 0–5 (0 = no fat, 1 = 1–5%, 2 = 6–33%, 3 = 34–66%, 4 = 67–100%, 5 = bulging), and documented if a brood patch or cloacal protuberance were present. Both males and females have brood patches [34]. A lack of a brood patch indicated that the individual was not breeding at the time of capture; it was not a reliable indicator of whether an individual bred at some time during the season. I weighed, measured, and banded young on the nest.

I attached 3.9 g (three percent of body weight) VHF radio transmitters (Advanced Telemetry Systems (ATS), Isanti, Minnesota, USA) to a subset of Clark’s nutcrackers with backpack harnesses. Due to logistical constraints, I did not randomly select birds to radio tag from among those captured.

#### Radio tracking

In 2010, I primarily triangulated the radio tagged Clark’s nutcrackers’ locations, and opportunistically attempted to home in on individuals. In 2011 and 2012, with the aid of field assistants, I primarily obtained point locations on Clark’s nutcrackers by homing. I attempted, when possible, to closely observe each radio tagged bird for a minimum of two hours each week, until the end of the field season (Table 2) or until an individual’s signal “disappeared”. If a signal was not heard, I continued to listen for it daily until I observed that the bird was alive, but had a broken antenna, or until the end of the field season. Antennas may have snapped due to preening; I observed individuals preening the antenna along with the feathers several times, and observed antennas which were curled rather than straight, likely due to preening. On eleven occasions, I searched for “missing” birds using dual wing-mounted H antennas attached to an airplane. During all observations while homing, I documented the activity budget (e.g. foraging, flying, perching, breeding activity), and habitat. I recorded all activities on initial observation of the individual, then continuously throughout the observation period, noting all times when the activity changed.

**Table 2:** Annual variation in documentation of population-wide breeding.

Year	Dates in field	# Trapped (Dates trapped)	# Radio tagged	Mean # days radio tagged birds observed	# Person-days (breeding and post-breeding seasons)
**2009**	**Mar 6—Nov 2**	**38 (Mar 24—Apr 20)**	**0**	**NA**	**102**
2010	Mar 7—Aug 19	15 (Mar 17—May 27)	13	4 ± 1	99
**2011**	**Jan 11—Nov 20**	**67 (Jan 28—Jun 27)**	**29**	**19 ± 2**	**327**
2012	Jan 7—Oct 31	35 (Jan 15—Mar 11)	34	17 ± 1	282
2013	May 19—Oct 1	0 (NA)	0	NA	157

Between years, there was variation in the dates and number of days I spent in the field, the number and dates of Clark’s nutcrackers trapped, the number radio tagged, and the number of days radio tagged birds were observed. Nonbreeding years are in bold.

I located nests by tracking radio tagged Clark’s nutcrackers to the nest and through incidental observations. By tracking radio tagged birds, I was able to observe each bird in all used habitats, and therefore habitat specific detectability did not play a role in locating radio tagged birds’ nests. I regularly monitored each active nest, each nest at which Clark’s nutcrackers were observed building, or where eggs or nestlings were present. I recorded the number of eggs and young, and estimated the age of young at each visit. When logistically feasible, I visited each nest after laying was complete to count the final clutch size, close to the estimated hatching date to determine the number of young hatched, between 13 and 17 days after hatching to band young, then between 22 and 28 days, the time period when fledging occurs (T. D. Schaming personal observation). In between, I opportunistically monitored nests when I followed radio tagged individuals to the site, or when I was close to the location. I considered young successfully fledged if I observed fledglings off the nest, likely fledged if the nest was empty within 22–28 days post-hatching, or fledging status unknown if nests were not checked within 22–28 days post-hatching, but were empty after 28 days. At all times while radio tracking, I recorded all observed fledglings and older young of the year.

I cannot reasonably compare proportion of breeding birds, number of breeding attempts, number of nests located, or number of fledged young between 2010 and 2012 because the sampling regimens were different. It is likely that I did not observe the majority of breeding attempts by radio tagged birds in 2010 because I did not home to individuals on a regular basis. However, in 2011 and 2012, it is unlikely that I missed breeding attempts because I routinely homed to individuals.

I tracked Clark’s nutcrackers using R410 digital scanning receivers (ATS), and a three element folding Yagi (ATS; AF Antronics, White Heath, Illinois, USA) or H (ATS) handheld antenna, and recorded the locations of individuals using portable global positioning system (GPS) units (Garmin International Inc., Olathe, Kansas, USA; Universal Transverse Mercator Zone 12N, NAD 1983).

#### Occupancy surveys

My protocol for establishing occupancy survey points evolved between years, based on experience and logistical constraints. In 2009, I located 48 points along eight 1 km transects. Transects were not set up on trails or roads. I grouped the points on transects so that I could walk between points in one day. The starting locations were randomly chosen from two focal areas within whitebark pine habitat, within sixty minutes of driving, and ninety minutes of hiking. Transect direction was randomly determined by spinning a compass rosette, avoiding paved roads, rivers and cliffs. To maintain spatial independence, each transect was spaced a minimum of 500 m apart, and each point on a transect was spaced 200 m apart, a standard distance for passerine point count surveys [35]. Though each starting location was in whitebark pine habitat, the ecosystem where I work is a mosaic of habitats. Habitats on the transects and traversed en route to the transects included all conifer habitats at the study site.

In 2011, I established 39 additional points within four focal areas. I located three focal areas at sites within a sixty minute drive of Jackson, WY; two had whitebark pine habitat within ninety minutes of a road, and one had only sparse whitebark pine habitat within 30 km. All six conifer habitats were represented in at least one focal area. I established 12, 12, and 10 random points within each area in conifer habitat, at least 400 m apart, using a random point generator (http://www.geomidpoint.com/random/). I also located two arbitrary points in the area with sparse whitebark pine habitat. I established three random points in a fourth focal area with whitebark pine, a 120 minute drive from Jackson, WY.

In 2012, I established nine additional points in three focal areas (four, three and two points) with whitebark pine as the dominant species. I randomly picked a starting point in each focal area, randomly picked the direction of the transect by spinning a compass rosette, then set up points 400 m apart. If blocked by a river, cliff, or other obstacle, I spun the compass again, and followed the first random direction greater than 45 degrees from direction of the last point. I also established seven arbitrary points in whitebark pine habitat near citizen science point count survey locations.

In 2013, to ensure the survey results were representative of the ecosystem, I established 134 additional points in locations throughout Bridger-Teton and Shoshone National Forests. I divided the two national forests into 30 equal sized focal areas. In 25 of the 30 focal areas (areas which did not already have six previously established points, were open to the public, and within ninety minutes from a road), I picked a random starting point in or within 2 km of whitebark pine habitat, then set up an additional two to five points, each 400 m apart, in a randomly chosen compass direction. I set up six, five, four or three points in seventeen, three, two and three areas. I also added ten additional points on two transects in a focal area established in 2012.

Each year, 2009 through 2013, I conducted point count surveys at newly established points and a subset of points established in previous years. During the surveys and while hiking to the survey locations, I recorded all observed fledglings and older young of the year.

#### Fledgling surveys

In 2012, I conducted fledgling surveys in two locations, one with a mosaic of all six conifer habitat types, and the second in Douglas-fir forest. I hiked in an arbitrary direction from the parking area, avoiding locations previously traversed, documenting the time and location of each fledgling seen or heard, the number of juveniles in a group, and the habitat at each siting.

#### Habitat classification

To determine habitat at survey points, I used a modified point quarter method at each survey location. From the primary survey point, then from four points 30 or 35 m to the northwest, northeast, southwest and southeast, I divided the area into four quadrants, along north-south and east-west axes. The distance of 30 or 35 m varied between years due to an error on the data sheets. In each quadrant at each of the five points, I documented the species of, and measured the circumference of and distance to the closest live tree, and the circumference of and distance to the closest live and dead whitebark pine tree. If no trees and/or no live and/or dead whitebark pine trees were present within 200 m, the quadrant was labeled as empty for that category.

To determine the habitat surrounding active nest or nest building trees, I estimated the proportion of the area within 100 m radius composed of each tree species. I did not conduct a point quarter method at six of the 247 occupancy survey points due to time constraints. I determined the habitat at these six points and at all other locations where I worked with a land cover type map in ArcGIS. I constructed a geospatial layer of land cover types using map data from the whitebark pine stand-level condition assessment [36], the Bridger-Teton National Forest (existveg_2007, USDA National Forest Service Remote Sensing Applications Center, obtained from Grand Teton National Park), Shoshone National Forest (FSVeg Spatial database, extracted March 22, 2012, obtained from U.S. Forest Service Rocky Mountain Region (R2) Regional Office, Geospatial Services), Grand Teton National Park (2005 vegetation mapping report, obtained from Grand Teton National Park), and Wyoming GAP analysis (U.S. Geological Survey Gap Analysis Program- Land Cover Data v2.2 [37]) vegetation maps. When discrepancies occurred, the layers were prioritized in the order listed. For the habitat at the six occupancy survey points at which I did not use a point quarter method, I also included qualitative habitat data on tree species present within 100 m of the point.

#### Environmental variables

Whitebark pine cone crop was represented by the average number of cones per tree documented by the Interagency Grizzly Bear Study Team’s annual cone counts throughout the region (Haroldson personal communication, [38]). Snowpack and temperature were represented by the average daily snow water equivalent (SWE), a measurement of the amount of water contained in the snowpack, and average daily temperature, respectively, during March, the beginning of the breeding season [39].

### Statistical Analyses

#### Breeding as a function of environmental variables

I used generalized linear mixed models (GLMMs) to test factors that were associated with breeding. I predicted that failure to breed would be linked to whitebark pine cone crop the previous autumn. Due to high early breeding season snowpack in the two nonbreeding years, I could not exclude the possibility that snowpack or an interaction between cone crop and snowpack was the driving factor for nonbreeding years. As snowpack is often related to temperature, I also examined the link between nonbreeding years and early breeding season temperature.

Due to the low power of a sample size of five years, I was unable to use one or separate GLMMs to evaluate the significance of whitebark pine cone crop, snowpack, temperature and the interaction between each as predictors for the probability of a breeding versus nonbreeding year. Therefore, to evaluate if nonbreeding years are related to cone crop versus snowpack or temperature, in three separate GLMMs, I evaluated whether a breeding or nonbreeding season significantly predicted the whitebark pine cone crop the previous autumn, snowpack, and temperature (zero-inflated negative binomial, Gaussian and Gaussian distributions, respectively). I included year as a random factor in each, and transect as a random factor in the model predicting whitebark pine cone crop.

#### Body condition index

Julian Date is known to affect mass of birds due to variation in food supply [40], and time of day captured is known to affect the mass of diurnal birds due to fasting overnight [41]. Therefore, to evaluate which variables to include in the adult body condition index, I first used a linear model to determine if mass (g) of adult Clark’s nutcrackers varied significantly with Julian date, time of day, or quadratic terms of each measure. I only included each individual the first time it was captured over all years (2009–2012). I then estimated relative body condition index as the residuals of body mass regressed against tarsus length, the body size indicator, and Julian date of capture, to correct for body size and date effects.

There is an ongoing debate in the literature on how to measure body condition of live animals [42,43]. The common technique of ordinary least squares (OLS) regression assumes the predictor variable is measured without error. I justify using OLS regression because the predictor variable, tarsus length, has negligible measurement error. First, only I measured each tarsus, eliminating a large source of potential measurement error between field workers. Second, repeatable measurements of the predictor variable increase its accuracy [44,45]. I measured the tarsus twice on each bird to ensure accuracy. In the rare case where measurements differed, I measured the tarsus a minimum of two additional times to ensure the readings were repeatable. A model II reduced major axis regression is recommended as an alternative to OLS regression; however, only one predictor variable is used in a reduced major axis regression [42]. To justify using OLS regression, I compared the OLS regression results of weight as a function of tarsus length only with a model II reduced major axis regression of the same variables. To evaluate if body condition index residuals significantly predicted fat score, I used a generalized linear model (GLM) with binomial distributions and a logit link function.

I used two-tailed t-tests to test the hypotheses that body condition index would differ between birds trapped during the prebreeding season in breeding versus nonbreeding years, and during the breeding season in breeding versus nonbreeding years. To evaluate if body condition index during the prebreeding or breeding season significantly predicted a breeding versus nonbreeding year, I used separate GLMs with binomial distributions and logit link functions.

To further examine the relationship between weight and Julian date of capture corrected for tarsus length, I conducted three additional linear models to determine if mass of adult Clark’s nutcrackers varied significantly with Julian date and tarsus length. One model included all years, the second only breeding years, and the third only nonbreeding years.

#### Body condition index as a function of environmental variables

To determine if the environmental factors associated with breeding also impact the body condition index, I used GLMMs to evaluate the significance of whitebark pine cone crop, snowpack, and temperature on the body condition index. I conducted three Kendall’s rank correlation tests to evaluate correlation between the three environmental variables. Due to the multicollinearity among the predictor variables, I used three separate GLMMs with Gaussian distributions and included year as a random factor in each.

#### Other

I used R (version 3.1.0) to perform all analyses. I checked for normality and homogeneity of variance, and met all key assumptions underlying application of GLMs and GLMMs. I applied p < 0.05 as the significance level, and report values as mean ± standard error of the mean. I include individuals recaptured between years in summary data in Tables 1 and 2, but only include an individual the first time it was captured in analyses.

### Data

All of my original data from which this article is based are deposited at Figshare http://dx.doi.org/10.6084/m9.figshare.1157837. Whitebark pine cone crop data was obtained from a third party, the Interagency Grizzly Bear Study Team (http://www.nrmsc.usgs.gov/research/igbst-home.htm), and is available upon request from Mark Haroldson (mark_haroldson@usgs.gov). Snowpack and temperature data are available online from the United States Department of Agriculture Natural Resources Conservation Service Togwotee Pass SNOTEL station (http://www.wcc.nrcs.usda.gov).

## Results

### Capture

My field assistants and I spent 1,109 person-days in the field, including 967 person-days during the breeding and post-breeding seasons (Table 2). Between 2009 and 2012, I trapped and banded 155 adult and 30 nestling Clark’s nutcrackers. Nine individuals were recaptured in subsequent years. I only observed brood patches on trapped Clark’s nutcrackers during the breeding season. Only including data from the first time each Clark’s nutcracker was trapped, individuals weighed an average of 130.1 ± 0.9 g (n = 140; range 106.4–155.6). The average tarsus length was 36.6 ± 0.1 mm (n = 146; range 33.3–38.9). The average culmen length was 38.3 ± 0.2 mm (n = 146; range 31.0–44.4). The average wing cord was 188 ± 1 mm (n = 146; range 173–201), and the average tail length was 118 ± 1 mm (n = 146; range 101–141). Variation in fat levels was very slight, and I only documented scores of 0 (n = 61) and 1 (n = 82). Body condition index residuals did not significantly predict fat levels (n = 143; β = 0.02 ± 0.02, p = 0.4).

### Radio tracking

I fit radio transmitters to, then regularly radio tracked 76 adults. In 2010, I only homed in on radio tagged Clark’s nutcrackers an average of 4 ± 1 days. On the other hand, in 2011 and 2012, I homed in on radio tagged individuals an average of 19 ± 2 and 17 ± 1 days, respectively. In 2010 and 2012, I found a total of 33 active nests (31 of radio tagged birds, 2 of unbanded birds). I observed six nest building activities for which I did not find a final nest (4 of radio tagged birds, 2 of unbanded birds). Three individuals were radio tagged in two different years, the first in 2010 then 2011 (no nesting observed in either year), the second in 2010 then 2012 (nesting observed in both years), and the third in 2011 then 2012 (nesting only observed in 2012). The habitat surrounding active nest or nest building trees (n = 32 locations measured) was composed of all six conifer species, whitebark pine, limber pine, Douglas-fir, lodgepole pine, Engelmann spruce, and subalpine fir at 53%, 9%, 25%, 47%, 44%, and 66% of the locations, respectively.

### Occupancy and fledgling survey habitat

Between 2009 and 2013, I conducted 1,066 thirty minute occupancy surveys at 247 point count locations (Fig 1). I carried out surveys in all six conifer habitats. I documented whitebark pine, limber pine, Douglas-fir, lodgepole pine, Engelmann spruce, and subalpine fir at 52%, 22%, 33%, 38%, 48% and 64% of the 247 survey points, respectively. In 2012, I conducted 73.7 hours of fledgling surveys (64.1 hours in a mosaic of all six conifer habitat types, and 9.6 hours in Douglas-fir forest).

### Habitats visited during daily field work

The study site was a mosaic of habitats, and while trapping, radio tracking, conducting occupancy and fledgling surveys, and hiking to survey locations, I regularly worked in all six conifer habitats at the site (Table 3).

**Table 3:** Percentage of days spent in each conifer habitat during the breeding and post-breeding seasons, seasons when it would have been possible to observe evidence of breeding.

Year	Whitebark pine (%)	Limber pine (%)	Douglas-fir (%)	Lodgepole pine (%)	Engelmann spruce (%)	Subalpine fir (%)
2009	70.6	26.5	60.8	49.0	64.7	94.1
2010	48.5	53.5	92.9	77.8	92.9	90.9
2011	52.3	44.0	85.0	85.3	79.2	86.9
2012	67.0	39.7	65.2	67.0	62.4	92.2
2013	72.0	46.5	62.4	71.3	77.7	83.4

Numbers do not add up to 100% because I spent time in multiple habitats every day.

### Determination of breeding versus nonbreeding years

During the five-year study, I did not observe any indications of individual Clark’s nutcrackers attempting to breed at our study site in two years, 2009 and 2011 (Table 4). On the other hand, in 2010, 2012 and 2013 I observed multiple indications of Clark’s nutcrackers breeding. We, however, did observe courtship behavior in 2010, 2011 and 2012, during both breeding and nonbreeding years, but only during the three years in which I radio tagged and regularly observed individual birds for long periods of time. Each spring, I contacted local bird watchers, personnel at six local wildlife-oriented nonprofits, the Wyoming Game and Fish Department, and Grand Teton Nation Park to request people contact me with observations of Clark’s nutcracker breeding behavior. In nonbreeding years, no-one had observed fledgling Clark’s nutcrackers, and no-one came forth with observations at a later date. However, in each of the three breeding years, local citizens emailed with anecdotal observations of Clark’s nutcracker fledglings. These observations were consistent with my determination of breeding versus nonbreeding years.

**Table 4:** Annual indications of Clark’s nutcracker breeding.

Year	Fledglings seen on study area	% Trapped adults with brood patches	# Radio tagged observed to attempt breeding	# Active nests observed	# Active nest building observations, final nest not found	Dates nesting activities observed
**2009**	**No**	**0% (0/38)**	**NA**	**0**	**0**	**NA**
2010	Yes	40% (6/15)	13% (2/13)	2	0	Mar 17-May 4
**2011**	**No**	**0% (0/67)**	**0% (0/29)**	**0**	**0**	**NA**
2012	Yes	6% (4/65)	88% (30/34)	31	6	Mar 5—Jun 15
2013	Yes	None trapped	NA	0	0	NA

Nonbreeding years are in bold.

In 2010, no young fledged from the observed nests (0/2). In 2012, young fledged from a minimum of 32% (10/31) and a maximum of 39% (12/31) of the active nests. I observed fledglings of radio tagged birds up to approximately 44 days after fledgling (n = 30 nestlings banded). During fledgling surveys, I observed one group (one or more fledglings together at one location) of fledglings every 8.2 hours (n = 73.7 hours; 0.12 ± 0.04 groups per hour).

### Breeding as a function of environmental variables

**Fig 2:**
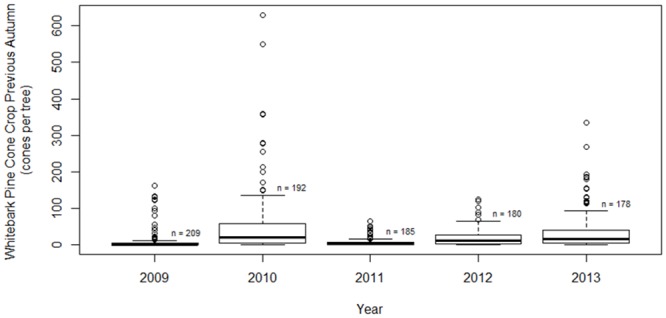
Whitebark pine cone crop in breeding versus nonbreeding years. Evidence suggests Clark’s nutcrackers did not breed population-wide in 2009 and 2011, years following low whitebark pine cone crops.

**Fig 3:**
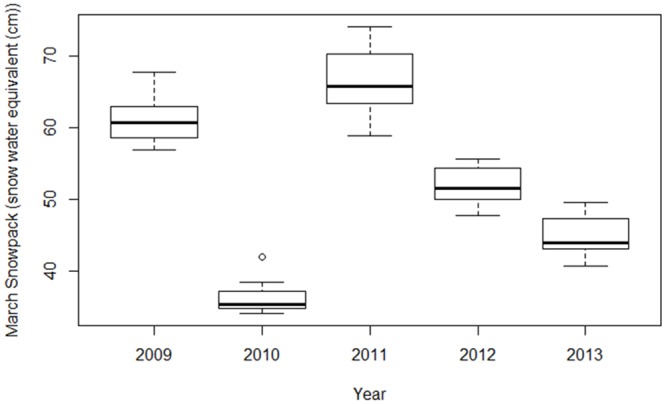
Snowpack in breeding versus nonbreeding years. Evidence suggests Clark’s nutcrackers did not breed population-wide in 2009 and 2011, years with high average March snowpack (n = 31 for all years).

Clark’s nutcrackers at the study site experienced large inter-annual differences in food availability and spring snowpack (Figs 2 and 3). The average whitebark pine cone crop was lower in the nonbreeding years (8.0 ± 1.7 and 5.2 ± 0.7 cones per tree) than the breeding years (46.5 ± 5.9, 19.8 ± 1.7 and 33 ± 3.7 cones per tree). However, the range of cones per tree (0–124) in 2012, a breeding year, fell within the range of cones in 2009, a nonbreeding year (0–161). Population-wide failure to breed was a significant predictor of a low cone crop the previous autumn (n = 944; β = 1.5 ± 0.3, p < 0.001), and a high average daily March snowpack (n = 155; β = -196.3 ± 62.6, DF = 3, p = 0.05), but did not statistically predict average daily March temperatures (n = 155; β = 1.3 ± 1.5, DF = 3, p = 0.45).

### Body condition index

I trapped Clark’s nutcrackers during four prebreeding seasons (2009–2012), two breeding seasons (2011–2012), and one post-breeding season (2011). Both the OLS and model II reduced major axis regressions of weight as a function of tarsus length showed a significant positive correlation, justifying the use of an OLS regression to determine body condition index (n = 140; β = 4.4 ± 0.9, p <0.001 and R^2^ = 0.14, slope = 17.2, angle = 86.7, p = 0.02, respectively). Over all years, mass decreased significantly with Julian date of capture (n = 140; β = -0.08 ± 0.03, p = 0.001). However, mass did not vary significantly with time of day captured, or the quadratic terms of each measure (p’s > 0.05). Therefore, I estimated body condition index as the residuals of body mass regressed against tarsus length and Julian date of capture, to correct for body size and date effects.

**Fig 4:**
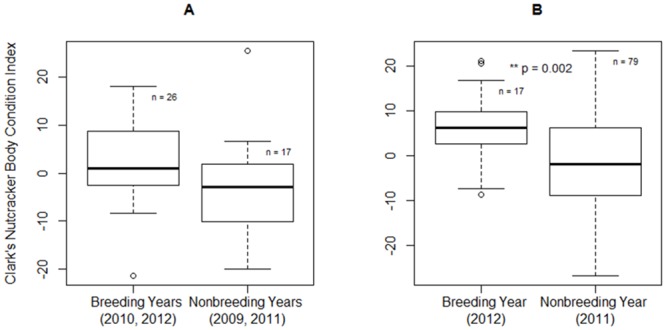
Clark’s nutcracker body condition index in breeding versus nonbreeding years. The Clark’s nutcracker prebreeding body condition index did not differ significantly between breeding and nonbreeding years (A). However, the average body condition index for birds during the breeding season was significantly higher in the breeding versus nonbreeding year (B). Body condition index is the residuals of body mass regressed against tarsus, corrected for date. Clark’s nutcrackers were trapped during four prebreeding seasons (2009–2012), but only during two breeding seasons (2011–2012).

Between breeding and nonbreeding years, adult prebreeding season body condition index did not differ significantly (n = 43; t = 1.8, df = 29.7, p = 0.09), and there was similar variability (n = 26, μ = 2.4 ± 1.7, range = -21.3–18.0, and n = 17, μ = -3.1 ± 2.6, range = -19.9–25.4, respectively; Fig 4). On the other hand, breeding season body condition index was significantly higher in the breeding versus nonbreeding year (n = 96; t = 3.4, df = 27.4, p = 0.002). However, there was higher variability in the nonbreeding year (n = 17, μ = 6.2 ± 2.0, range = -8.8–21.1, and n = 79, μ = -1.5 ± 1.1, range = -26.7–23.3, respectively; Fig 4). During the breeding season, some individuals during the nonbreeding year had a body condition index as high as those from the breeding year. Lower adult body condition index during the prebreeding season did not predict a nonbreeding year (n = 43; β = 0.06 ± 0.04, p = 0.08). In contrast, lower body condition index during the breeding season significantly predicted a nonbreeding year (n = 96; β = 0.08 ± 0.03, p = 0.007).

**Fig 5:**
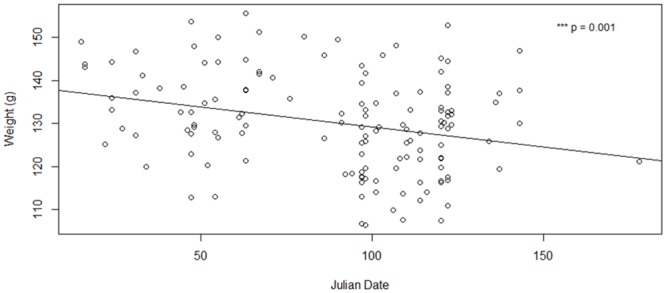
Clark's nutcracker weight variation over the trapping season (2009–2012). The weight of trapped Clark’s nutcrackers decreased significantly with Julian date of capture.

Over all years, weight increased significantly with tarsus (n = 140; β = 4.12 ± 0.88, p <0.001) and decreased significantly with Julian date of capture (n = 140; β = -0.08 ± 0.03, p = 0.001; Fig 5). When examined separately, during breeding and nonbreeding years weight increased significantly with tarsus (n = 43; β = 3.8 ± 1.3, p = 0.006 and n = 97; β = 4.1 ± 1.1, p <0.001, respectively). During breeding years, though there was a decreasing trend, weight did not decrease significantly with Julian date (n = 43; β = -0.05 ± 0.04, p = 0.2). However, during nonbreeding years, weight did not vary with Julian date (n = 97; β = -0.002 ± 0.04, p = 0.96). The significance of Julian date over all years, but not during breeding and nonbreedings years separately, is possibly due to the dates of trapping differing during breeding and nonbreeding years (Table 2). The average Julian date of capture was 61 ± 5 (n = 43 birds, range = 15–136) and 102 ± 3 (n = 97 birds, range = 33–178) in breeding and nonbreeding years, respectively.

### Body condition index as a function of environmental variables

I determined body condition index for 140 Clark’s nutcrackers. The whitebark pine cone crop, average daily March snowpack, and average daily March temperature experienced by the individuals were multicollinear. Cone crop was correlated with March snowpack (z = -13.7, p < 0.001) and March temperature (z = 8.9, p < 0.001). March snowpack and temperature were correlated (z = -8.9, p < 0.001). Clark’s nutcracker body condition index was not predicted by whitebark pine cone crop the previous autumn, average daily March snowpack, or average daily March temperatures (n = 140; β = 0.2 ± 0.1, DF = 2, p = 0.2; β = -0.3 ± 0.2, DF = 2, p = 0.2; and β = 1.8 ± 1.6, DF = 2, p = 0.4, respectively).

## Discussion

During two of the five years of the study, I had strong evidence suggesting that the Clark’s nutcrackers did not breed population-wide in any of the diverse conifer habitats within my study area. I did, however, observe Clark’s nutcracker courtship displays in both breeding and nonbreeding years. Clark’s nutcracker courtship displays, as in many avian species, occur throughout the year, likely as a means of reinforcing pair bonds [17,46]. Therefore, it is not inconsistent that courtship was observed in nonbreeding years. Elucidation of the specific ecological triggers and exact causal mechanisms underlying absence of breeding will require long-term studies. However, as predicted, nonbreeding years followed autumns with low whitebark pine cone crops. This result is confounded by high average March snowpack in the two nonbreeding years. Consequently, I cannot separate the effects of cone crop and snowpack. Low whitebark pine cone crop, high snowpack, or an interaction between the two, may have influenced the population-wide nonbreeding. They may have provided environmental cues available to the birds prior to breeding which allowed them to predict that breeding conditions would be poor due to low food, leading to the decision to skip breeding. On the other hand, they may have resulted in low body energy stores, leading all individuals to choose not to or be unable to breed. Clark’s nutcrackers are long-lived birds, living up to 17 years in the wild [47]. Therefore, adaptively skipping reproduction during resource poor years is consistent with life history theory and maximization of lifetime reproductive success [3,4].

It is possible that Clark’s nutcrackers may shift their habitat selection in years with high versus low whitebark pine cone crops, and behavior may vary in different habitats. However, I eliminated bias in assessing breeding behavior within the population by regularly observing Clark’s nutcracker behavior in all six conifer habitats present with my study area each year. Clark’s nutcrackers are known to irrupt in years with cone crop failure [20,21], and a portion of the local population may have emigrated from the study area due to low whitebark pine cone crop. However, a number of Clark’s nutcrackers did stay in the study area each year. During breeding years, I located nests in areas which contained all conifer types. Because I observed nutcracker behavior in each conifer habitat each breeding and post-breeding season, I should not have missed evidence of Clark’s nutcracker breeding in any of the six conifer habitat types during nonbreeding years due to biased sampling.

Whitebark pine is an important Clark’s nutcracker food source in the Greater Yellowstone Ecosystem. The two other primary foods, limber pine and Douglas-fir were unlikely to have played a significant role in breeding decisions. Limber pines at the site were few and patchy. Though they are an important late summer food source, the majority of seeds are eaten immediately rather than cached (T. D. Schaming personal observation). On the other hand, the Douglas-fir cone crop was consistently high throughout the study site each year (T. D. Schaming personal observation). However, despite Clark’s nutcrackers regularly foraging on Douglas-fir, the seeds contain much less nutrition than whitebark pine seeds (0.06 versus 1.19 kcal per seed, respectively; [48,49]). Clark’s nutcrackers are estimated to require 11,827 kcal to survive from mid-October through mid-April, when alternative foods become available [26]. Therefore, an individual requires 9,939 whitebark pine seeds or 197,117 Douglas seeds to survive each winter. Clark’s nutcrackers cache substantially more seeds because some may spoil or will escape through germination, rodents may steal caches, some caches may become inaccessible or may not be relocated, and some may be needed to feed young [26]. Consequently, though Douglas-fir are more numerous, the increased handling time per seed (24.3 versus 4.9 s/seed for whitebark pine [16,49]) and quantity of seeds they would require to survive the winter, it is unlikely that they could replace whitebark pine in the diet.

Barringer et al. [50] determined that Clark’s nutcrackers visit whitebark pine stands with lower cone production less frequently, resulting in a lower probability of seed dispersal in such stands. A low whitebark pine cone crop would lead to both fewer seeds available to be cached and lower visitation by Clark’s nutcrackers. The Clark’s nutcrackers would therefore have less food, fewer cached whitebark pine seeds, to feed themselves and their young over the winter and following spring. Clark’s nutcrackers slowly lose their memory of their seed caches after 183 days, and many seeds remaining after the first year would have germinated, spoiled or been robbed by other animals. Thus, they would not be able to rely on cached seeds from years other than the most recent autumn [19]. Due to fewer seed caches, the birds may also have been forced to travel farther to find alternative food if adequate supplies of cached seeds were not available on their breeding territories. This may not have been possible if they needed to return regularly to a nest.

Snowpack may also play a role in Clark’s nutcrackers skipping breeding. Snowpack may have made it difficult to retrieve seeds cached underground or to forage for other types of supplementary food. However, Clark’s nutcrackers do retrieve caches from under the snow (T. D. Schaming personal observation, [51]). They cache up to 59% of seeds aboveground [52], and regularly cache in exposed areas, such as steep cliffs and south facing slopes, where wind and sun prevent heavy snow accumulation [26,29,49]. Even during the springs with high snowpack, I regularly observed Clark’s nutcrackers using locations with both bare, exposed slopes and deep snowpack.

To my knowledge, there is no evidence that high snowpack prevents Clark’s nutcrackers from nesting in a given year. High snowpack seems more likely to influence when the birds can start breeding, rather than their overall tendency to skip breeding [51]. Nest building dates from populations in diverse geographical locations vary from mid- to late January in British Columbia through June 1 in the eastern Sierra Nevada, CA [51,53]. In fact, Clark’s nutcrackers are primarily found in high, mountainous areas [17], and nests have been found in locations with deep snow (e.g. [18,54]). I regularly observed Clark’s nutcrackers during the breeding season in habitats with high snowpack. Between years, I often observed individuals in the same, snow-covered locations regardless of the interannual variation in snowpack.

Previous authors have suggested the Clark’s nutcrackers may forego breeding in years with low food, but this is the first study to positively document it [18,26]. Foregoing breeding may be an adaptive strategy of Clark’s nutcrackers to face the trade-off between survival and reproduction owing to environmental constraints. Skipping breeding in poor years, years with low resources, such as food or breeding sites, may lead to a higher likelihood of future breeding or survival to a later year. If so, breeding plasticity could be one means for Clark’s nutcrackers to maximize lifetime reproductive success while exploiting a variable environment. Such an adaptive strategy, skipping breeding in resource-poor years, is not uncommon among vertebrate species. Pinyon Jay (*Gymnorhinus cyanocephalus*) breeding is only predictable following major cone crops, and some or all do not attempt to nest in the spring after a local cone crop failure, whereas Crossbills (genus *Loxia*) are believed to breed only when there is a high seed supply [9,55]. Dormice (*Glis glis*) stay sexually inactive when beech trees are not masting [56]. Snow petrels (*Pagodroma nivea*) will not breed when severe snow and ice impede access to nesting locations [57], and red-footed boobies (*Sula sula*) are more likely to skip breeding in El Niño years when high sea surface temperatures reduce food supplies [7].

Skipping breeding may be an effective adaptive strategy to face the survival-reproduction trade-off. However, if poor environmental conditions which lead to skipping breeding become more prevalent, individuals may skip breeding more often than in the past. Such an increase could lead to a population decline. Because some habitats may historically have had enough good resource years to maintain populations, individuals may continue to prefer the habitats after the number of poor-resource years increases. This would lead to an ecological trap [58]. If the population-wide failure to breed is caused by low whitebark pine cone crops and Clark’s nutcrackers stay in or near the whitebark pine habitats without breeding, the declining whitebark pine habitats may become sink habitats for the birds [59].

**Fig 6:**
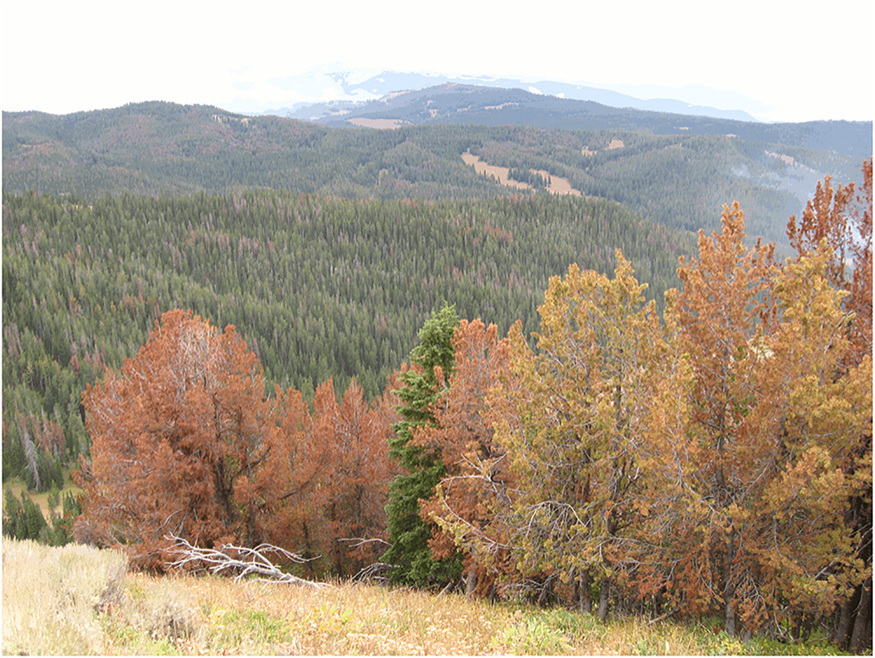
Dying whitebark pine trees. Example of whitebark pine trees at the study site which are dying due to mountain pine beetle attacks the previous year. (Photo credit: T. D. Schaming).

Whitebark pine forest communities are rapidly disappearing range-wide, and even some of the healthiest whitebark pine stands, located in the Greater Yellowstone Ecosystem, have severely declined since 1999 (Fig 6) [24]. The decline is primarily due to a mountain pine beetle epidemic that has been worsened by favorable effects of global warming on bark beetle reproduction [60]. In 2009, 46% of these whitebark pine stands were classified as “high mortality” [61]. Clark’s nutcrackers regularly experience significant inter-annual differences in food availability because the whitebark pine is a masting conifer; however, because the whitebark pine is declining, the number of whitebark pine seeds available during both masting and nonmasting years is lower. Years with low whitebark pine cone crops occur frequently in the Greater Yellowstone Ecosystem: 44% (15/34) of the years since 1980 had cone crops at or lower than the average levels of the 2009 and 2011 nonbreeding years. Since 1980, 24% (8/34) of the years had a high March 1st snowpack, a level at or higher than the average levels observed in the 2009 and 2011 nonbreeding years. Fifteen percent (5/34) of the years since 1980 had both low cone crops and high snowpack.

Whitebark pine cone crop, high snowpack or an interaction with cone crop and snowpack may be the driving force behind the decision to forego breeding. If so, the continuing decline of whitebark pine trees, and the predicted increase in number of years with extreme snow, could have a strong negative effect on stability of the regional Clark’s nutcracker population [62]. The proportion of potential breeders that actually breeds affects population growth rate. Therefore, factors which influence the number of breeders can act as a strong regulatory mechanism. Nonbreeding years would have important effects on population size and structure. It is possible that the extreme years in which all individuals in a population fail to breed have more important effects on population size and age structure than “average” years [63,64]. As stated by Anderson et al. [65], “Understanding how patterns of behavior change as landscapes are altered through time may provide important insights into mechanisms underlying observed demographic trends in populations”.

An increase in the number of Clark’s nutcracker nonbreeding years could also have serious ecosystem-wide consequences. Clark’s nutcrackers shape the ecosystems in which they live: annually, individuals store tens of thousands of seeds in thousands of separate locations [29,49]. Seeds not retrieved for food are able to germinate [33]. Clark’s nutcrackers disperse seeds up to 32.6 km, rapidly and effectively moving seeds longer distances than wind, rodents and all other North American seed hoarding birds. They enable rapid migration of seeds, and contribute to gene flow across and between habitat islands [52]. They often move seeds across latitude and elevation, as well as into disturbed areas [52,66]. Clark’s nutcrackers may cache the majority of seeds in areas unsuitable for germination [52]. However, because individuals cache such a high volume of seeds each year, they “plant” many seeds in in microhabitats and local landscapes suitable for germination and establishment [52]. In the face of current climate and habitat change, the long-distance dispersal of conifer seeds, and thus the continued association between Clark’s nutcrackers and conifers, may be critical in mitigating against local genetic bottlenecks and inbreeding depression. Such dispersal bolsters effective population size and facilitates rapid colonization of newly available ideal habitats.

In ecosystems with increased variability resulting from climate and habitat change, individuals could have a higher probability of encountering poor resource conditions; such conditions would result in a lower probability of successful reproduction and survival [4]. Habitat specialists in particular may be especially vulnerable to such environmental changes if they are unable to adapt. Accurately predicting the impact of both declining habitat and a more variable climate is a major challenge in ecology [4]. It is important to understanding the ecological triggers and exact causal mechanisms of population-wide decisions not to breed; in the face of climate change and environmental disturbance, this information will increase our ability to effectively manage populations and communities.
